# CITN11-02 interim trial results: subcutaneous administration of recombinant human IL-15 (rhil-15) is associated with robust expansion of peripheral blood CD56+ NK cells

**DOI:** 10.1186/2051-1426-2-S3-P80

**Published:** 2014-11-06

**Authors:** Chihiro Morishima, Douglas G McNeel, Manish R Patel, Holbrook Kohrt, Thomas A Waldmann, John A Thompson, Kevin Conlon, Paul M Sondel, Heather Wakelee, Mary L Disis, Stephen P Creekmore, Jeffrey S Miller

**Affiliations:** 1 University of Washington, Seattle, WA, USA; 2 University of Wisconsin, Madison, WI, USA; 3 University of Minnesota, Minneapolis, MN, USA; 4 Stanford University, Stanford, CA, USA; 5 Lymphoid Malignancy Branch/Center for Cancer Research/National Cancer Institute, Bethesda, MD, USA; 6 Division of Cancer Treatment and Diagnosis, National Cancer Institute, Frederick, MD, USA

## Background

IL-15 activates and induces the proliferation of CD8+ T cells and NK cells. The Cancer Immunotherapy Trials Network (CITN) is conducting a Phase I, open-label, dose-escalation study of subcutaneous (SQ) rhIL-15 in advanced melanoma, renal cell, non-small cell lung and squamous cell head and neck carcinoma patients. The primary objective is to determine the maximum tolerated dose; secondary objectives include evaluation of immunological activity defined by increases in circulating lymphocytes.

## Methods

Each cycle consists of 5 daily SQ injections of rhIL15 (E.coli-derived, NCI) administered Monday-Friday for two weeks, followed by 2 weeks observation. The absolute lymphocyte count is evaluated daily during the 10 days of SQ injection and whole blood flow cytometric analysis of T and NK cell numbers is conducted on Days 1 and 11 of each cycle.

## Results

Three patients have been enrolled in each of the 0.25, 0.5, 1.0 and 2.0 mcg/kg/dose cohorts (n = 12). Ten patients have completed 2 or more cycles and two have completed one. Only one serious adverse event, grade 2 pancreatitis, was observed in a metastatic melanoma patient and began 3 days after completing Cycle 1 treatment at 2.0 mcg/kg. Flow cytometric data indicate a consistent increase in the frequency of CD3-CD56+ NK cell numbers at Day 11 compared to Day 1 of Cycle 1 (mean 3.6-fold increase, range 0.7-8.1). Notably, the subpopulation of CD56^bright ^NK cells increased 6.7-fold (mean, range 1.8-17.9). Increases in CD56+ and CD56^bright ^NK cell frequencies were less pronounced in Cycle 2 (mean fold-increase = 1.6 and 2.9, respectively) and in subsequent cycles. The percentage of CD56+ NK cells among CD45+ cells was higher on Day 11 (mean = 20, range 9-32%) compared to Day 1 of Cycle 1 (mean = 8, range 3-22%). Two patients demonstrated remarkably high percentages of CD56+ NK cells peaking at 42-43% of CD45+ cells. By marked contrast, the frequency of CD8+ T cells was largely unchanged during Cycle 1 (mean fold-increase = 1.2, range 0.5-2.9) and subsequent cycles.

## Conclusion

SQ rhIL-15 was very well tolerated through the 2 mcg/kg/dose, associated with an increase in CD56+ NK cells, and a substantial expansion in the CD56^bright ^NK cell subpopulation. The effect on peripheral blood T cells was surprisingly minimal. The 2 mcg/kg/dose cohort will be expanded to six patients before dose escalation proceeds. After defining the optimal dosing regimen, combinations with appropriate monoclonal antibodies will be of interest.

This study was supported by NIH 1U01 CA154967-01 (http://clinicaltrials.gov/ NCT01727076).

